# The METTL3/miR-196a Axis Predicts Poor Prognosis in Non-small Cell Lung Cancer

**DOI:** 10.7150/jca.92968

**Published:** 2024-01-21

**Authors:** Zhen Yang, Jie Hao, Minghan Qiu, Ruxue Liu, Hanwei Mei, Qiaonan Zhang, Zhanhua Gao, Wenwen Pang, Jing Liu, Wenjie Pan, Huaqing Wang, Ming Gao

**Affiliations:** 1 Department of Clinical Laboratory, Tianjin Union Medical Center of Nankai University, Tianjin 300121, China.; 2 The Institute of Translational Medicine, Tianjin Union Medical Center of Nankai University, Tianjin 300121, China.; 3 Department of Oncology, Tianjin Union Medical Center of Nankai University, Tianjin 300121, China.; 4 Department of Thyroid and Breast Surgery, Tianjin Key Laboratory of General Surgery in Construction, Tianjin Union Medical Center of Nankai University, Tianjin 300121, China.

**Keywords:** METTL3, m6A, miR-196a, GAS7, NSCLC, immune cell infiltration

## Abstract

**Background:** METTL3 accelerates m6A modification to influence cancer progression including non-small cell lung cancer (NSCLC). To illustrate the role and underlying mechanism of METTL3 mediated miR-196a upregulation in NSCLC.

**Method:** The global level of m6A modification was detected by qPCR, western blot and immumohistochemical staining. The TCGA, GEPIA, CPTAC and TIMER databases were used to explore the expression change of METTL3, miR-196a and GAS7 in NSCLC patients. Kaplan-Meier analysis was performed to analyze the prognostic value of miR-196a. NSCLC cells overexpressed or knockdown miR-196a were constructed and used for CCK8, colony formation assay, western blot and immunofluorescence in vitro. The effect of miR-196a on tumor growth was investigated in vivo.

**Result:** We found that METTL3 mediated miR-196a were notably enhancive in NSCLC tissues and in NSCLC cells, which is markedly positively related with the serious TNM stage, the large tumor size, the distant metastasis, and the poor prognosis in patients of NSCLC. Further investigation showed that up-regulated miR-196a promoted cell viability and cell autophagy, while down-regulation of miR-196a revealed opposite results in H1299 and A549 cells. In terms of mechanism, we found that miR-196a interacted with GAS7. In addition, GAS7 expression in NSCLC patients may be positively related with the infiltration of immune cell subsets in tumor microenvironment (TME).

**Conclusion:** The axis of METTL3-miR-196a-GAS7 might be a target for molecular targeted therapy, a potential and novel diagnostic marker for NSCLC patients.

## Introduction

Lung cancer is one of the most common malignancies, resulting in approximately 1.6 million deaths every year. Non-small cell lung cancer (NSCLC) is the major subtype of lung cancer (approximately 85% of lung cancer cases), including lung squamous cell carcinoma and lung adenocarcinoma, which is the leading cause of cancer death worldwide 1. Lung cancer is a primary cancer type with high morbidity and mortality in China 2. Surgery and chemotherapy are the main treatment modality for NSCLC 3. Recent advances with targeted therapies and immunotherapy improved outcomes by targeting identifiable driver oncogenes in a small number of patients with NSCLC 4. However, most NSCLC tumors respond poorly to treatment and the overall survival rate of patients with NSCLC remains poor. Understanding lung cancer-related regulatory factors and exploring the molecular mechanism of this cancer has important significance in providing early detection, diagnosis, and treatment of lung cancer.

The methyltransferase-like (METTL) family is a group of different methyltransferases that methylate nucleotides, small molecules and proteins. The most typical family members, METTL3 and METTL14, dimerize to form N6-methyl ladenosine (m6A) RNA methyltransferase, which has an established role in cancer progression 5. More and more evidence suggested that abnormal METTL3-mediated m6A levels are involved in the malignant progression of lung cancer through the different molecular mechanisms, including angiogenesis, invasion, proliferation, metastasis, glycolysis, drug resistance, tumor environment and cancer stem cells 6-10. For example, METTL3 can promote the expression of AKT1 protein by regulating the m6A level of AKT1 mRNA, thus promoting the NSCLC progression and chemotherapy resistance 11. In addition, Jiang et al reported that METTL3 improved the stability of methylated lnc-SNHG1 transcripts by reducing the rate of RNA degradation, thus leading to the upregulation of lnc-SNHG1 in NSCLC. They found that the METTL3/lnc-SNHG1 /miRNA-140-3p axis may regulate the expression of UBE2C and then contributes to progression of NSCLC 12. Recently, Li et al reported that METTL3 silencing increased the level of pri-miR-663 and m6A methylated pri-miR-663, and inhibited the maturation of miR-663 and the expression of miR-663. METTL3 promoted tumor growth by regulating the miR-663/SOCS6 axis in vivo 13. However, whether METTL3 regulates miR-196a in NSCLC is unclear.

Based on the findings above, METTL3 was hypothesized to be involved in NSCLC by regulating the m6A methylation of miR-196a in this study, which gives us insight into new therapeutic targets for NSCLC.

## Material and Methods

### Clinical samples

Tissues of 32 patients with NSCLC and adjacent normal tissues were collected from December 2011 to November 2014. All NSCLC patients received initial surgery treatment. The inclusion and exclusion criteria are listed as follows: The inclusion criteria were: (1) postoperative pathology was NSCLC; (2) patients with complete clinical data. The exclusion criteria were: (1) patients combined with other lung cancer pathology; (2) patients combined with other malignant tumors; (3) patients receiving chemotherapy or radiotherapy or other treatment before surgery. The current study was conducted with the approval of the Ethics Committee of Tianjin Medical University Cancer Institute and Hospital (Ek2019034), and all participants gave their written consent.

### Cell culture

BEAS-2B, H1299, A549, H57 and A973 cells were purchased from the National Infrastructure of Cell Line Resource (Beijing, China), which were cultured in DMEM (Corning, Beijing, China) with 10% FBS (Gibco, Carlsbad, CA, USA), 100 μg/ml streptomycin (Gibco, Carlsbad, CA, USA), 100 U/ml penicillin (Gibco, Carlsbad, CA, USA) in 5% CO2 at 37°C. shR-METTL3, pri-miR-196a, Anti-miR-196a, pEGFP-GAS7 3'UTR WT or MUT and the empty vector or the negative control were transferred into cells using Lipofectamin 3000 (Invitrogen, Carlsbad, CA, USA).

### RT-qPCR assay

Total RNAs were extracted from tissues and cells using TRIzol Reagent (Invitrogen, Carlsbad, CA, USA). Reverse transcription was carried out using the RT-PCR Quick Master Mix Kit (TOYOBO CO., LTD. Life Science Department OSAKA JAPAN). The resultant cDNA was amplified using a SYBR® Green Realtime PCR Master Mix (TOYOBO CO., LTD. Life Science Department OSAKA JAPAN). The sequences of PCR primers are listed. pri-miR-196a-qPCR-S: 5'GAGGCGTCAGTTTCTTTGGTC3'; pri-miR-196a-qPCR-AS: 5'TAGCGTAAAAGGAGCAACATAGT3'; U6-qPCR-S: 5'TGCGGGTGCTCGCTTCGGCAGC3'; U6-qPCR-AS: 5'CCAGTGCAGGGTCCGAGGT3'; METTL3-qPCR-S: 5'CGTACTACAGGATGATGGCTTTC3'; METTL3-qPCR-AS: 5'TTTCATCTACCCGTTCATACCC3'; GAPDH-qPCR-S: 5'AAGGTGAAGGTCGGAGTCAAC3'; GAPDH-qPCR-AS: 5'GGGGTCATTGATGGCAACAATA 3'.

### CCK8 assay

Briefly, 24 hours before transfection, cells were plated into 96-well plates at a density of 3×10^3^ cells in 100 μl medium per well. During the 48 h of culture, each well was added with 10 μl CCK8 reagent (Beyotime, Shanghai, China) and incubated at 37℃ for 1 h. The relative cell proliferation rate was detected using a microplate reader (Thermo Fisher, Ltd., CA, USA) at absorbance of 450 nm.

### Colony-formation assay

Briefly, the cells were plated in 12-well petri dishes with a density of 500 cells per dish and cultured in a 5% CO2 incubator at 37°C. Change the medium every 3 days. After 10 weeks, the cells were immobilized with methanol for 20 min and stained with 0.5% crystal violet for 10 min (Beyotime, Shanghai, China) and the colonies formed were counted manually.

### Bioinformatics analysis

The METTL3 and GAS7 mRNA and protein level was analyzed by GEPIA (http://gepia.cancer-pku.cn/) and UALCAN (http://ualcan.path.uab.edu/); The miR-196a level was analyzed by OncomiR (http://www.oncomir.org/); The survival curve of LUAD and LUSC patients was analyzed by Kaplan Meier plotter (http://kmplot.com/); The immune cell infiltration in LUAD and LUSC patients was analyzed by TIMER (https://cistrome.shinyapps.io/timer/).

### Western blot

The total proteins in tissue samples and cells were extracted using RIPA lysis buffer (Beyotime, Shanghai, China), boiled at 100°C for 5 min, and centrifuged at 12,000 rpm/min for 5 min. The concentration of protein was determined using the BCA Kit (Thermo Fisher, Ltd., CA, USA). Supernatants were separated on 10% SDS-PAGE and transferred onto PVDF membranes (Millipore, USA) and blocked with 5% non-fat milk (Solarbio, Beijing, China). Then, the protein samples were incubated at 4˚C overnight with METTL3 (1:1500, SRP13070, Tianjin Saier Bio), GAPDH (1:3000, SRP00849, Tianjin Saier Bio), LC3 (1:1500, SRP01707, Tianjin Saier Bio), BECN1 (1:2000, ab207612, Abcam), p62 (1:2000, SRP01795, Tianjin Saier Bio) and GAS7 (1:500, 10072-1-AP, Proteintech Bio). After 1xTBST washing, the protein samples were incubated with secondary antibodies at room temperature (1:2000, #7074, CST) for 1 h. Finally, ECL kit (Beyotime, Shanghai, China) was used to assess protein bands.

### IF staining

A549 cells were washed with cold 1xPBS (Dingguo Bio, Beijing, China) and then fixed with 4% paraformaldehyde (Biosharp, Beijing, China) at room temperature for 30 minutes. After being washed thrice with cold 1xPBS (Dingguo Bio, Beijing, China), the cells were blocked in 10% BSA (Beyotime, Shanghai, China) at room temperature for 10 min. The cells were subsequently incubated with primary antibodies specific for LC3 (SRP01707, Saier Biotechnology, Tianjin, China) at 4°C overnight. The next day, the cells were washed with cold 1xPBS (Dingguo Bio, Beijing, China), after which they were incubated with fluorescence-conjugated secondary antibodies (A0562, Beyotime, Shanghai, China) at room temperature for 1 h, followed by DAPI (C1002, Beyotime, Shanghai, China) at room temperature for 5min. Images were captured under a confocal microscopy.

### Tumor xenograft model

5×10^5^ A549 cells of the indicated groups were injected into the right subcutaneous tissue of nude mice for tumor transplantation. The subcutaneous length and width of the tumor were measured every 7 days during the 28 days in vivo transplantation trial. After 28 days, the mice were killed. All animal experiments were performed with the approval of the Ethics Committee of Tianjin Medical University Cancer Institute and Hospital (LLSP2019-017).

### Statistical analysis

GraphPad Prism V (GraphPad Software, Inc.) was used for image editing and SPSS V19.0 (IBM Corp.) Statistical software was used for data analysis. Measurement data were expressed as the mean ± SD and compared using the unpaired t-test. P<0.05 was considered to indicate a statistically significant difference.

## Results

### M6A modification and METTL3 is up-regulated in NSCLC

**Figure 1:**
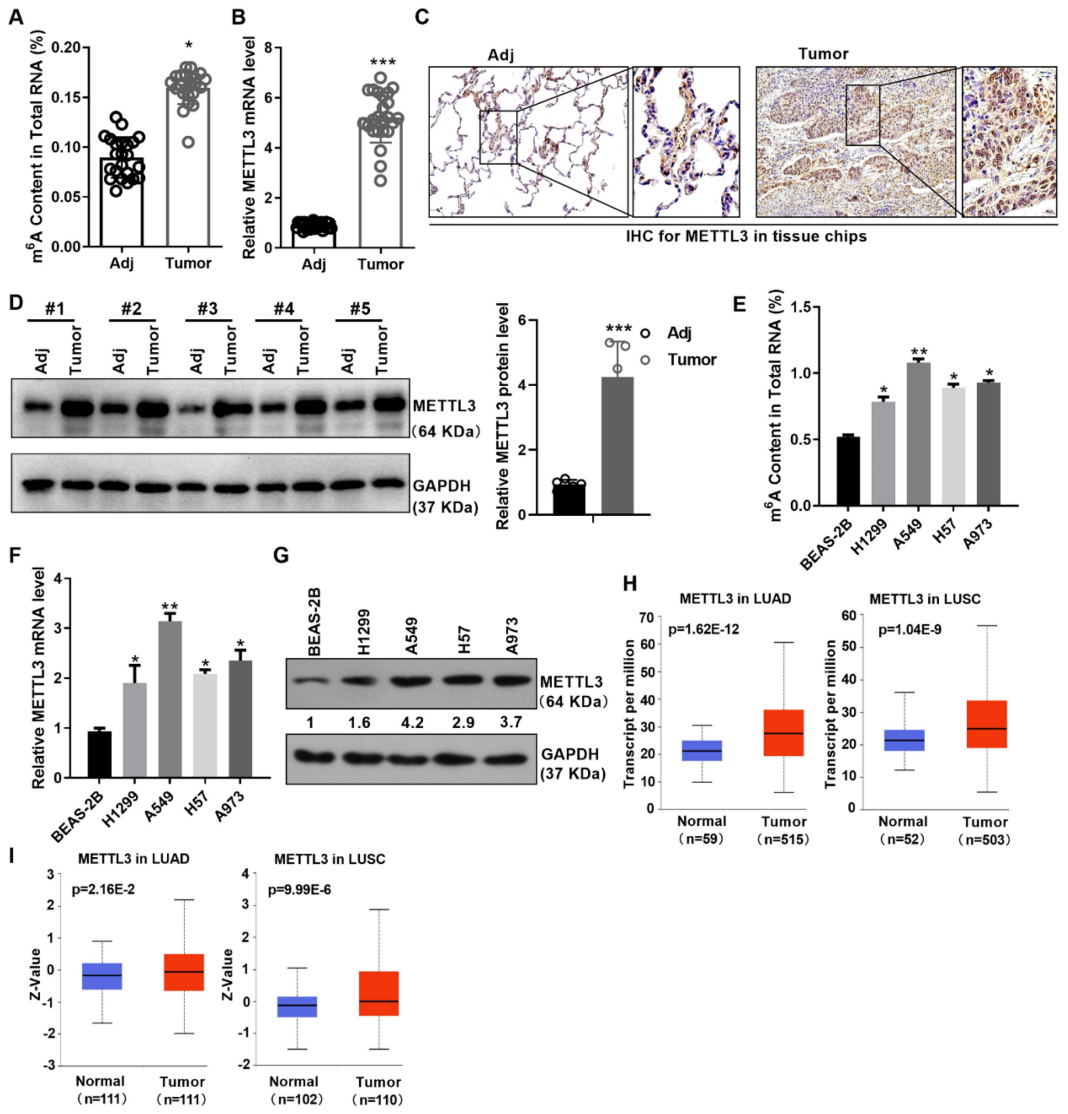
** m6A modification and METTL3 is up-regulated in NSCLC.** (A) m6A content in NSCLC tissues and the adjacent tissues was detected by m6A RNA Methylation Assay. (B) METTL3 mRNA level in NSCLC tissues and the adjacent tissues was detected by qPCR. (C) METTL3 protein level in NSCLC tissues and the adjacent tissues was detected by IHC. The tissue chip was from Biomax. (D) METTL3 protein level in NSCLC tissues and the adjacent tissues was detected by western blot. (E) M6A content in NSCLC and the immortalized lung epithelium cells was detected by m6A RNA Methylation Assay. (F) METTL3 mRNA level in the indicated cells was detected by qPCR. (G) METTL3 protein level in the indicated cells was detected by western blot. (H) TCGA data showed the mRNA level of METTL3 in LUAD and LUSC. (I) CPTAC data showed the protein level of METTL3 in LUAD and LUSC. Data are presented as means ± SD.*P<0.05; **P<0.01; ***P<0.001; ns, not significant.

To illustrate the role of m6A modification in NSCLC, we first measured the m6A levels in the adjacent normal tissues and NSCLC tissues. The results indicated that the m6A levels were obviously increased in NSCLC tissues compared with the adjacent normal tissues (Figure 1A). To better understand the expression and localization of METTL3 protein in non-small cell lung cancer, we monitored the mRNA and protein levels of METTL3 in NSCLC tissues and adjacent tissues using qPCR, immumohistochemical staining (IHC) and western blot assay. Results showed that the expression of METTL3 was significantly up-regulated in the tumor tissues of NSCLC (Figure 1B-1D). Furthermore, METTL3 was mainly localized in the cytoplasm and nucleus by IHC assay (Figure 1C). In addition, we also found that m6A modification and METTL3 expression is up-regulated in cell lines of NSCLC at mRNA and protein levels (Figure 1E-1G). What is more, according to the TCGA and CPTAC database, METTL3 was highly expressed in lung squamous carcinoma (LUSC) and lung adenocarcinoma (LUAD) at mRNA and protein levels (Figure 1H and 1I), suggesting its role as an oncogene.

### METTL3 mediated the m6A methylation promoted the miR-196a level

**Figure 2:**
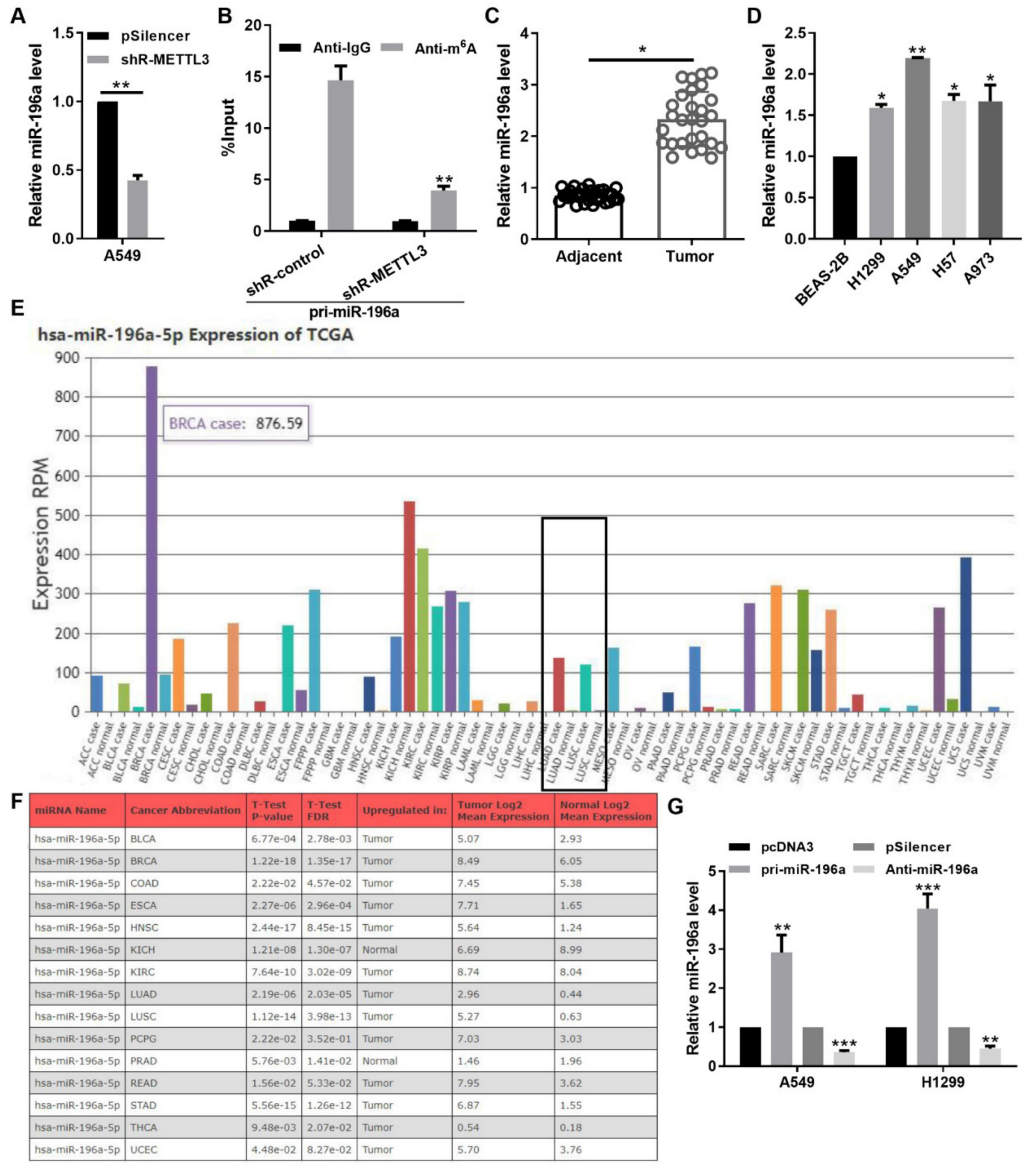
** METTL3‑dependent m6A methylation promoted the miR-196a level.** (A) qPCR showed the miR-196a level in A549 cells regulated by shR-METTL3. (B) m6A modification level of pri-miR-196a in A549 cells treated with shR-METTL3 detected by MeRIP assay. (C) qPCR showed the miR-196a level in NSCLC tissues and the adjacent tissues. (D) qPCR showed the miR-196a level in the indicated cells. (E) TCGA data showed the miR-196a level in many cancers. (F) OncoMIR data showed the miR-196a level in the indicated cancers. (G) qPCR showed the miR-196a level in A549 and H1299 cells. Data are presented as means ± SD.*P<0.05; **P<0.01; ***P<0.001; ns, not significant.

Due to the addition of the m6A marker is a key post-transcriptional modification that regulates the miRNA biogenesis, we next measured the effect of METTL3 loss on the miR-196a level. METTL3 knockdown reduced the expression level of miR-196a through the qPCR assay (Figure 2A). Moreover, the level of miR-196a m6A modification was markedly decreased following the konckdown of METTL3 in A549 cells (Figure 2B). To further identify the pathological and prognostic significance of miR-196a in NSCLC, the expression of miR-196a in NSCLC samples and cell lines were quantified using qPCR. The results showed that the miR-196a level is obviously augmented in NSCLC samples and NSCLC cell lines compared with the control groups (Figure 2C and 2D). In addition, the data from TCGA and OncomiR showed that miR-196a is up-regulated in many cancers, including NSCLC (Figure 2E and 2F). To determine the role of miR-196a in NSCLC cells, A549 and H1299 cells were infected with pri-miR-196a or anti-miR-196a and the control vector (Figure 2G).

### miR-196a overexpression facilitated cell proliferation and autophagy

**Figure 3:**
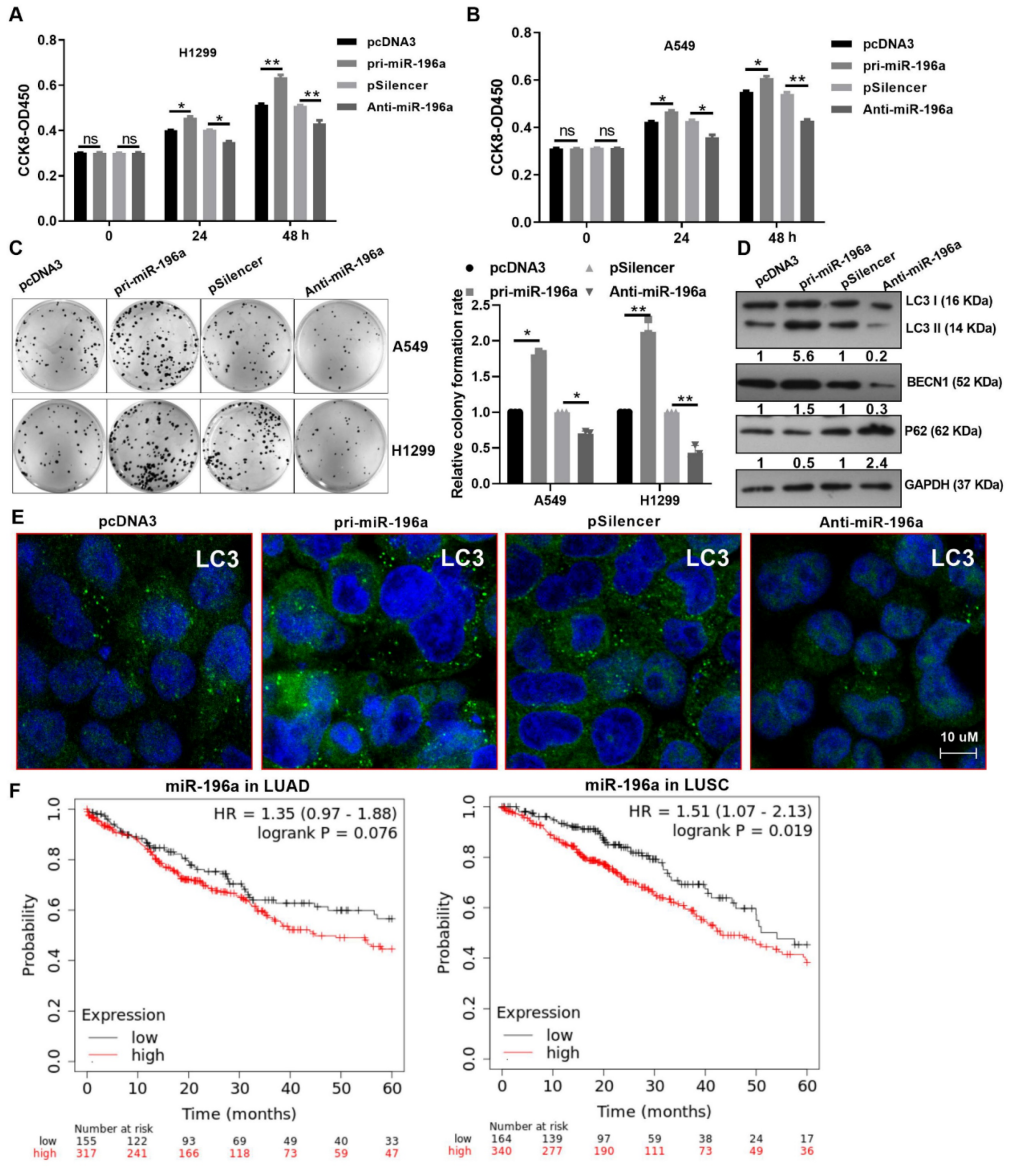
** miR-196a promoted cell proliferation and autophagy.** (A, B) CCK8 assay showed the cell viability of H1299 and A549 cells transfected with pri-miR-196a or Anti-miR-196a. (C) The proliferation ability of H1299 and A549 cells transfected with pri-miR-196a or Anti-miR-196a were detected by colony formation assay. (D) The indicated protein levels were detected by western blot in A549 cells transfected with pri-miR-196a or Anti-miR-196a. (E) The LC3 distribution and expression was detected by IF in A549 cells. (F) Kaplan Meier plotter showed the overall survival of patients with high or low expression of miR-196a in LUAD and LUSC. Data are presented as means ± SD.*P<0.05; **P<0.01; ***P<0.001; ns, not significant.

Overexpression of miR-196a facilitated cell viability compared with the control group, and anti-miR-196a inhibited cell viability in A549 and H1299 cells by CCK8 assay (Figure 3A and 3B). Similarly, pri-miR-196a and Anti-miR-196a remarkably enhanced or reduced the colony formation ability in H1299 and A549 cells compared with the control groups (Figure 3C). Several studies have indicated that the activation of autophagy contributes to the proliferation, whether or not miR-196a regulates autophagy is not clear. Here, we found that pri-miR-196a promoted but anti-miR-196a inhibited the protein levels of LC3 and BECN1, pri-miR-196a inhibited but anti-miR-196a promoted the protein levels of p62, the autophagy related genes (Figure 3D).

An immunofluorescence assay for LC3 showed that pri-miR-196a significantly increased autophagic puncta and anti-miR-496a decreased autophagic puncta in A549 cells (Figure 3E). These data demonstrated that miR-196a can heighten autophagy and promote proliferation in NSCLC cells. Furthermore, the expression of miR-196a was positively correlated with distant metastasis, TNM stage, and tumor size, (Table 1). that Patients with high miR-196a expression had significantly worse survival in LUAD and LUSC patients by the Kaplan-Meier analysis (Figure 3F).

### miR-196a directly target and decrease GAS7 expression

**Figure 4:**
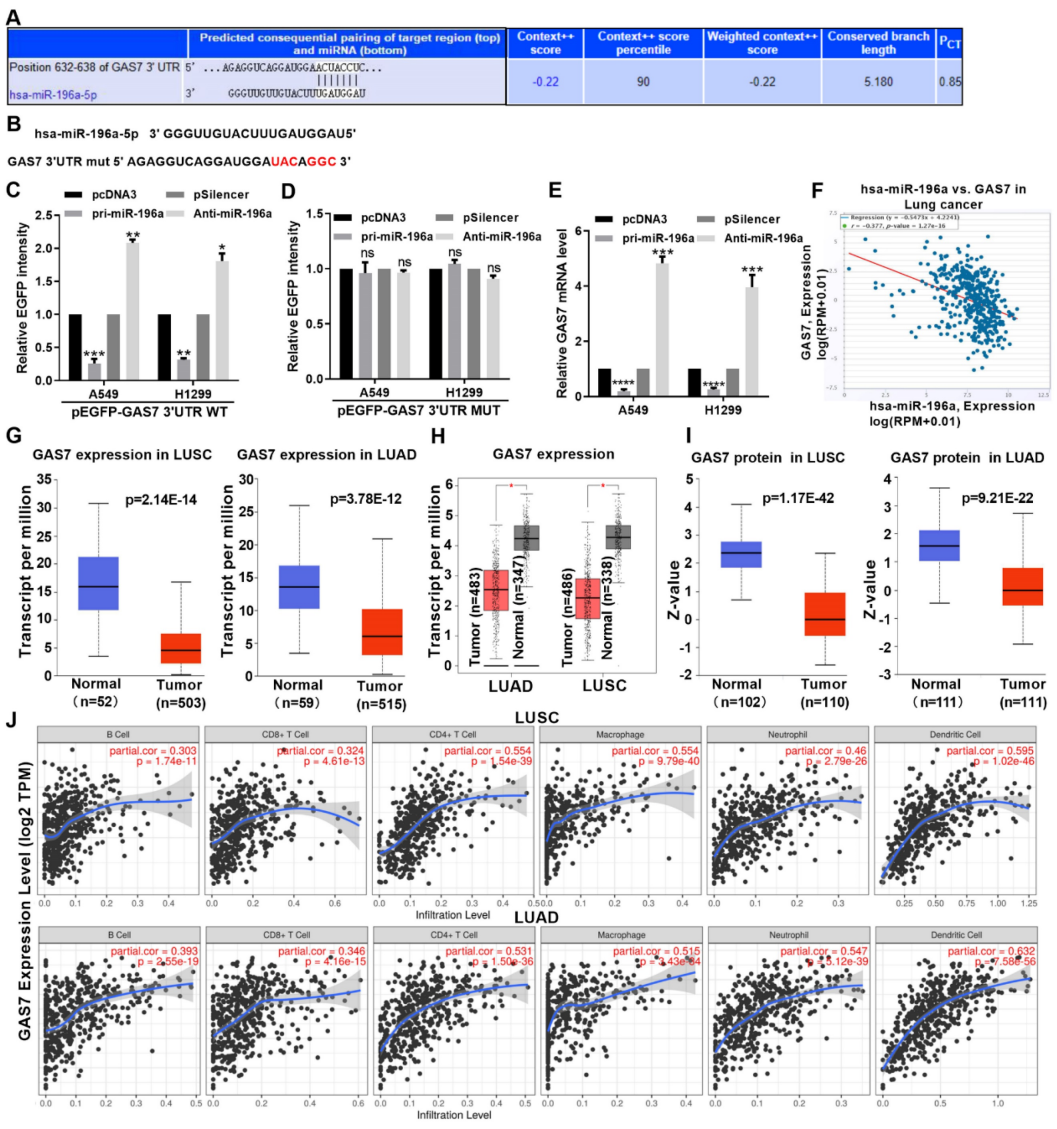
** miR-196a directly target GAS7.** (A) Predicted miR-196a binding sites in GAS7 mRNA 3' UTR showed through TargetScan. (B) The sites of GAS7 mRNA 3' UTR MUT was shown. (C, D) EGFP intensity of A549 and H1299 cells co-transfected with pri-miR-196a or Anti-miR-196a and the wild type or mutated 3'UTR of GAS7. (E) GAS7 mRNA expression levels with indicated transfection were measured by qPCR in A549 and H1299 cells. (F) ENCORI database showed the relationship of miR-196a and GAS7 in NSCLC. (G) TCGA data showed the mRNA level of GAS7 in LUAD and LUSC. (H) GEPIA data showed the mRNA level of GAS7 in LUAD and LUSC. (I) CPTAC data showed the protein level of GAS7 in LUAD and LUSC. (J) TIMER database showed the immune cell infiltration in LUAD and LUSC. Data are presented as means ± SD.*P<0.05; **P<0.01; ***P<0.001; ns, not significant.

miRNAs are known to induce gene silencing by binding to the 3 'untranslated region of targeted mRNA. To further investigate the promoting tumorous role of miR-196a, we used TargetScan to predict the functional target gene of miR-196a. This analysis showed that GAS7 may be a target of miR-196a in NSCLC (Figure 4A). An EGFP reporter plasmid containing GAS7 3′UTR and GAS7 3′UTR MUT was constructed and used to co-transfect cells with miR-196a (Figure 4B). miR-196a transfection significantly decreased the EGPF activity of GAS7 3′UTR but not the GAS7 3′UTR MUT. Anti-miR-196a transfection significantly increased the EGPF activity of GAS7 3′UTR but not the GAS7 3′UTR MUT (Figure 4C and 4D). According to the results of the experimental report, miR-196a overexpression significantly reduced GAS7 mRNA levels compared to transfection of both anti-miR-196a and scrambled control (Figure 4E). At the same time, our findings also suggested an inverse correlation between GAS7 and miR-196a by ENCORI database (Figure 4F). Then, the results of TCGA and GEPIA showed that GAS7 mRNA had lower expression in LUSC and LUAD tissues (Figure 4G and 4H). In addition, GAS7 protein was also decreased in LUSC and LUAD tissues by CPTAC database (Figure 4I). Furthermore, we found that the GAS7 expression was positively related to the infiltration of CD8 T cells, B cells, macrophages, CD4 T cells, neutrophils and Dendritic cells in the tumor microenvironment of NSCLC patients (Figure 4J).

### miR-196a promotes the growth of tumors in vivo

**Figure 5:**
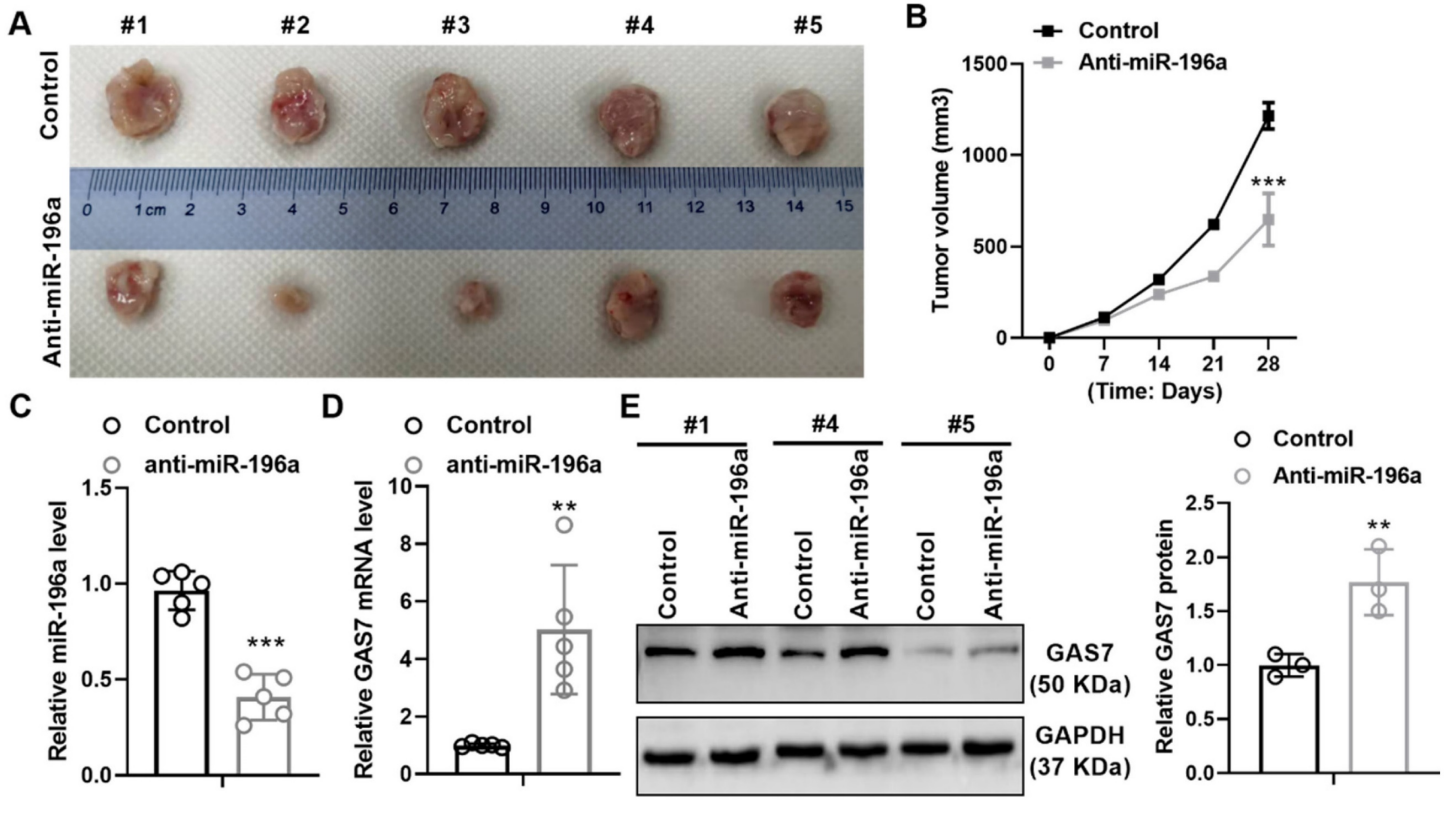
** miR-196a promotes tumor growth in vivo.** (A) The tumor size of the Anti-miR-196a and the Control group. (B) The tumor growth rate in the indicated time. (C) qPCR showed the level of miR-196a in tumor tissues. (D) qPCR showed the mRNA level of GAS7 in tumor tissues. (E) Western blot assay showed the protein level of GAS7 in tumor tissues. Data are presented as means ± SD. **P<0.01; ***P<0.001.

To further identify the promoting role of miR-196a, we hypodermically injected A549 cells stably treated with a knockdown plasmid of miR-196a or control into BALBc-nude mice to produce xenografts. Stable expression of anti-miR-196a lead to the suppressive tumor growth in tumor size and volumes compared with the control group (Figure 5A and 5B). In addition, we detect the level of miR-196a and GAS7 in tumor tissues and found that miR-196a is obviously downregulated and GAS7 is markedly up-regulated in anti-miR-196a group (Figure 5C and 5D). Western blot assay showed that the GAS7 protein levels were also up-regulated in tumor tissues of Anti-miR-196a (Figure 5E). These data indicated the negative relationship of miR-196a and GAS7 in tumor tissues.

## Discussion

New evidence suggests that miRNAs play a key role in a variety of biological functions in many diseases and dysfunction of miRNAs are intimately related to tumors, especially in NSCLC 14-17. miR-196a is obviously enhancive in many types of tumors and involved in a variety of biological processes biological processes via mRNA cleavage and translational inhibition, such as cell cycle, migration, proliferation, and apoptosis, mostly functioning as an oncogene 18-20. In lung cancer, forced expression of miR-196a alone induces cell proliferation and inhibits apoptosis by down-regulating GLTP, increasing cell resistance to gefitinib therapy in vitro and in vivo 21. In addition, miR-196a promotes the development and progression of NSCLC by down-regulating GPX3 and activating the JNK pathway 22. Liu et al reported that the expression of miR-196a could be regulated by DNA demethylation, and the high expression of miR-196a is associated with the higher clinical stage, as well as the lymph node metastasis of NSCLC 23. However, it is not clear whether miR-196a is regulated by m6A modification and whether miR-196a is involved in regulating autophagy in NSCLC. This study identified the miR-196a promoted proliferation of A549 and H1299 cells and autophagy in NSCLC cells and miR-196a promoted the growth of tumors in vivo, which is positively related to the distant metastasis, the TNM stage and the tumor size.

METTL3, a major regulator of RNA m6A methylation, has been shown to be oncogenic in various cancers 24. Recent findings have revealed that METTL3 is remarkably associated with different aspects of lung cancer progression, influencing the prognosis of patients 25. In this study, we also found that METTL3 is up-regulated in NSCLC tissues and cell lines by qPCR and TCGA database, indicating the carcinogenic effect of METTL3 in NSCLC. The m6A methylation process mediated by METTL3 affects the different stages of mRNA metabolism and the biological production of long non-coding RNAs, circRNAs and miRNAs in many cancers 26. Bioinformatics analyses were performed for circQSOX1 expressions, specific binding sites, and N6-methyladenosine (m6A) motifs of circQSOX1 and found that METTL3-mediated RNA m6A modification on circQSOX1 could be read by IGF2BP2 in CRC cells 27. In addition, METTL3 mediated m6A modifications facilitate miR-25-3p maturation in an m6A-dependent manner 28. In NSCLC, Pan et al reported that the METTL3/YTHDF2 m6A axis regulates the expression of miR-1915-3p through the transcription factor KLF4, and miR-1915-3p significantly inhibits migration and invasion and epithelial-stromal transition. 29. Here, we identified that METTL3 can promote miR-196a expression in cells, which is consistent with the work of other researchers in CRC indicating the up-regulation of miR-196b regulated by METTL3 30.

Found by predictive database analysis, GAS7 was predicted as a potential target gene of miR-196a. Then, the EGFP reporter assay confirmed that miR-196a can directly interact with GAS7 3'UTR to inhibit GAS7 expression in NSCLC cells. Therefore, our results suggest that miR-196a enhances its anticancer effect by directly targeting GAS7.

The GAS7 gene is located on the chromosome 17p 31. Dong et al reported that through whole genome sequencing and SNP array analysis of primary tumor samples, 17p chromosome is frequently deleted in high-risk neuroblastoma tumor subsets 32. In the acute myeloid leukemia, Wu et al reported that GAS7 overexpression played the tumor suppressive role and obviously reduced the expression levels of PCNA, CDK4 and cyclin D1 33. GAS7 may be involved in the pathogenesis of schizophrenia by regulating neurite growth and neuronal migration through its C-terminal F-BAR domain 34. In breast cancer, GAS7 was associated with CYFIP1 and WAVE2 complex to suppress breast cancer metastasis via blocking CYFIP1 and Rac1 protein interaction, actin polymerization, and β1-integrin/FAK/Src signaling 35. Ping et al reported that GAS7 is a direct target gene of miR-181a, and high levels of GAS7 mRNA are associated with improved overall survival 36. In this study, we found that miR-196a can directly target GAS7 and the expression of GAS7 was markedly downregulated, which is negatively correlated with the expression of miR-196a in NSCLC patients, indicating the suppressive effect of GAS7 in NSCLC.

## Conclusion

We summarized a new pathway in METTL3 regulated miR-196a directly targeting GAS7 on cell growth and autophagy. Our experimental data also suggested that the METTL3/miR‐196a/GAS7 axis may be a promising therapeutic target for NSCLC or other cancers.

## Figures and Tables

**Table 1:** Association of miR-196a with the clinicopathological features

Variables	No. cases (32)	miR-196a expression		*p*-value
		Low (n=14)	High (n=18)	
Age				
<60 years	11	6	5	0.728
≥60 years	21	8	13	
Gender				
Male	16	6	10	0.722
Female	16	8	8	
Tumor size				
≥5 cm	17	4	13	0.031^a^
<5 cm	15	10	5	
TNM stage				
I-II	12	9	3	0.035^a^
III-IV	20	5	15	
Distant metastasis				
No	15	10	5	0.031^a^
Yes	17	4	13	
Histological type				
Squamous	13	6	7	0.821
Adenocarcinoma	19	8	11	

**a**χ^2^ test. P-values in bold print indicate statistically significant differences.
